# The dissection-based identification of the preaortic sympathetic plexus formation, anatomical relations, and clinical applications

**DOI:** 10.25122/jml-2025-0069

**Published:** 2025-04

**Authors:** Mihaly Enyedi, Georgian-Theodor Badea, Radu-Tudor Ion, Daniela Elena Gheoca Mutu, Stefan Oprea, Zoran Florin Filipoiu

**Affiliations:** 1 Discipline of Anatomy, Department 2 – Morphological Sciences, Carol Davila University of Medicine and Pharmacy, Bucharest, Romania; 2 Doctoral School, Carol Davila University of Medicine and Pharmacy, Bucharest, Romania; 3 Faculty of Medicine, Carol Davila University of Medicine and Pharmacy, Bucharest, Romania

**Keywords:** preaortic plexus, lumbar splanchnic nerves, celiac ganglion

## Abstract

The abdominal sympathetic nervous system provides sympathetic innervation to the abdominal organs and gonads. This system is part of an extensive neural network that extends from the base of the skull to the pelvis. The preaortic (or prevertebral) plexus is a key component of the abdominal sympathetic system and is represented by a variable nervous network located anterior to the abdominal aorta. The aim of our study was to identify all these sympathetic structures and describe the formation and relationships of the preaortic plexus. We examined five cadavers (aged 66–71) with no medical or surgical history, preserved in 9% formalin at the Anatomy Department from Carol Davila University. Regional dissections were performed in successive planes, highlighting the major abdominal plexuses, the lumbar splanchnic nerves, and the associated network of neural connections that contribute to the preaortic plexus. The plexus is formed by efferent fibers from the celiac and aortico-renal ganglia, as well as from the three lumbar splanchnic nerves. The lumbar splanchnic nerves originate in the paravertebral sympathetic chains. We identified all these sympathetic structures and described the formation and anatomical relationships of the plexus. The nerve fibers of various origins form a longitudinally oriented network located anterolateral to the abdominal aorta. The lower part of this network continues into the superior hypogastric plexus. This neural network is delicate, complex, and variable, making it challenging to identify anatomically and surgically. Situated deeply in the retroperitoneal space, it is prone to accidental injuries during surgery in this compartment.

## INTRODUCTION

The autonomic nervous system is a fundamental component of the peripheral nervous system responsible for regulating involuntary physiological functions, such as cardiovascular activity, digestion, respiration, and glandular secretion. It is traditionally divided into the sympathetic and parasympathetic systems, which work together to maintain homeostasis by adapting organ function to the body's changing needs. The sympathetic division, often associated with the 'fight or flight' response, mobilizes energy resources and enhances alertness in response to stress, while the parasympathetic division promotes rest, digestion, and recovery.

Within the sympathetic nervous system, specific regional networks control different organ systems. Among these, the abdominal sympathetic nervous system plays an important role in autonomic regulation by providing sympathetic innervation to the abdominal organs and gonads. The abdominal sympathetic nervous system is part of an extensive neural network that extends from the base of the skull to the pelvis, integrating visceral functions and maintaining homeostasis. This system consists of prevertebral and paravertebral ganglia interconnected by nerve fibers that facilitate communication between the central nervous system and the target organs. The abdominal portion includes major sympathetic ganglia, such as the celiac, superior mesenteric, and inferior mesenteric ganglia, which contribute to the modulation of digestive, renal, and reproductive functions. Through this intricate network, the sympathetic nervous system influences processes such as gastrointestinal motility, vascular tone, and endocrine secretion, ensuring a coordinated physiological response to various internal and external stimuli [1].

A fundamental structural component of the abdominal sympathetic network is the prevertebral aortic plexus, a complex nerve network located in front of the abdominal aorta that serves as a major conduit for sympathetic fibers traveling to the abdominal viscera [2]. This plexus integrates input from the sympathetic ganglia and distributes sympathetic visceral afferent fibers to the abdominal organs [3].

Structurally, the plexus is composed of efferent fibers originating from the celiac and aortico-renal ganglia, as well as from the three lumbar splanchnic nerves [3], which arise from the paravertebral sympathetic chains [1]. These fibers form an interconnected longitudinal network positioned anterolateral to the abdominal aorta [4]. As the plexus extends inferiorly, its lower portion continues into the superior hypogastric plexus, ensuring continuity of sympathetic innervation toward the pelvic organs [5]. Several secondary plexuses emerge from the prevertebral aortic plexus, including the superior mesenteric, renal, gonadal, and inferior mesenteric plexuses, which further distribute autonomic input to their target organs.

This study aimed to identify all these sympathetic structures and the formation and relationships of the plexuses through detailed cadaveric dissection.

## MATERIAL AND METHODS

The research was conducted on five cadavers—three men and two women, aged between 66 and 71 years, without any medical or surgical history. The cadavers were preserved using a 9% formaldehyde solution in the dissection laboratory of the Anatomy Department at Carol Davila University of Medicine and Pharmacy in Bucharest.

The dissections were performed layer by layer, closely following the steps of surgical procedures. Dissections were performed in such a manner to resemble both an anterior and a posterior approach to the retroperitoneal compartment, aiming for the complete exposure of the abdominopelvic sympathetic chain, the identification of associated nerve plexuses, and the examination of their connections with visceral structures. The access route will be specified under each figure in the results section. Various surgical instruments, such as scalpels, forceps, scissors, tweezers, and retractors from the department’s toolkits, were used in this study.

The results were documented through photographs with a semi-professional digital single-lens reflex (DSLR) camera (Canon EOS 2000D with a standard 18-55 mm lens and an LED photo light kit), with a particular focus on the prevertebral aortic plexus. The obtained raw images were digitally processed to enhance clarity using Adobe Lightroom version 8.2.1(2019) software without altering their scientific content. The labels on the images were added using the Adobe Illustrator software version 27.2(2023).

## RESULTS

**Figure 1:**
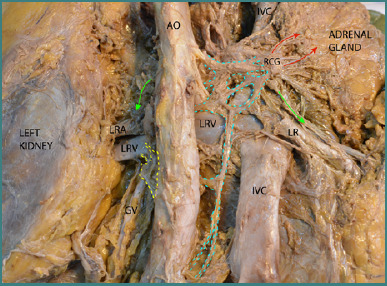
Dissection of the celiac ganglia, aorta, and inferior vena cava from a posterior approach. Legend: AO, Aorta; IVC, Inferior Vena Cava; RCG, Right Celiac Ganglion; LRV, Left Renal Vein; LRA, Left Renal Artery; GV, Gonadal Vein. Arrows and Lines: Red Arrows – Efferent fibers from the celiac ganglion to the adrenal plexus; Green Arrows – Efferent fibers directed toward the right and left kidneys; Blue Dotted Line – Efferent fibers from the celiac ganglion to the prevertebral aortic plexus; Yellow Dotted Lines – Left aortico-renal ganglion.

In Figure 1, the dissection was performed through a posterior approach to the celiac region and the major vessels. In the midline, the posterior surface of the aorta is visible, with the posterior surface of the inferior vena cava located to its right. The right celiac ganglion is posterior to the inferior vena cava, positioned between the aorta and the right adrenal gland.

As observed, the ganglion exhibits radially oriented efferent fibers (hence the region's designation as the solar plexus in the works of Testut) [6]. These efferent fibers extend toward the right adrenal gland, the aorta, and the renal arteries and inferiorly toward the prevertebral aortic plexus.

The left celiac ganglion is partially visible, covered in this figure by the abdominal aorta. Small groups of efferent fibers emerge from this ganglion and head to the left aortico-renal ganglion. The left aortico-renal ganglion can be seen in the angle between the left renal vessels and the abdominal aorta.

**Figure 2:**
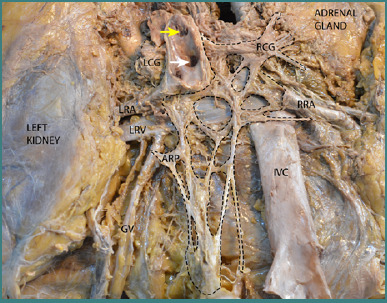
Dissection through a posterior approach of the celiac ganglia and inferior vena cava after the removal of the aorta, illustrating the formation of the prevertebral aortic plexus. Legend: LRA, Left Renal Artery; LRV, Left Renal Vein; GV, Gonadal Vein; IVC, Inferior Vena Cava; RCG, Right Celiac Ganglion; LCG, Left Celiac Ganglion; ARP, Aortico-Renal Plexus; RRA, Right Renal Artery (cut). Arrows and Markers: Yellow Arrow – The origin of the celiac trunk; White Arrow – The origin of the superior mesenteric artery; Inside the Dotted Line – The preaortic plexus and the left and right celiac ganglia.

In Figure 2, after removing the aorta, it can be observed that fiber groups originating from the two celiac ganglia converge anteriorly to the aorta, forming the prevertebral aortic plexus. This plexus also includes fibers from the aortico-renal ganglia. Although the dense network of nervous fibers that forms the prevertebral aortic plexus can be easily noticed in this figure, greater consideration should be given to the particularly close relationships between the nervous components and the vascular structures in the retroperitoneum. It is these relationships that make surgery at this level challenging and potentially risky.

In a separate dissection, a systematic exploration of the abdominal cavity was conducted, with all anatomical layers carefully traversed to expose the anterior aspect of the retroperitoneal compartment. Particular attention was given to preserving the integrity of the anatomical structures while ensuring optimal visualization. The secondary retroperitoneal viscera, including the pancreas and the duodenum, were not excised; instead, they were gently retracted toward the left side to facilitate a clearer view of the underlying structures.

**Figure 3:**
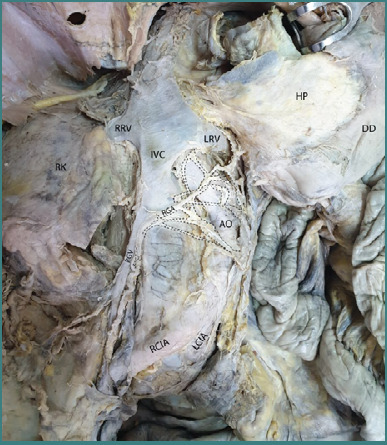
Anterior approach of the retroperitoneal compartment highlighting the origin of the sympathetic nerve plexus surrounding the right gonadal artery. Legend: AO, Abdominal Aorta with kinking; RCIA, Right Common Iliac Artery; LCIA, Left Common Iliac Artery; RGV, Right Gonadal Vein; RGA, Right Gonadal Artery; IVC, Inferior Vena Cava; RRV, Right Renal Vein; LRV, Left Renal Vein; DD, Descending Duodenum (retracted); HP, Head of Pancreas (retracted); RK, Right Kidney. Inside the black dotted line – The prevertebral plexus, from which branches emerge and run along the right gonadal artery.

As illustrated in Figure 3, the prevertebral aortic sympathetic plexus can be observed descending along the anterior surface of the abdominal aorta. Notably, the aorta exhibited a leftward convexity in this specimen, a morphological variation consistent with aortic kinking. The complex network of preaortic nerve fibers was distinctly visible, contributing to the formation of fine branches that extended toward the gonadal artery, forming a perivascular autonomic plexus. Such an anatomical configuration emphasizes the intricate relationship between the autonomic nervous system and the vascular structures within the retroperitoneal space.

**Figure 4:**
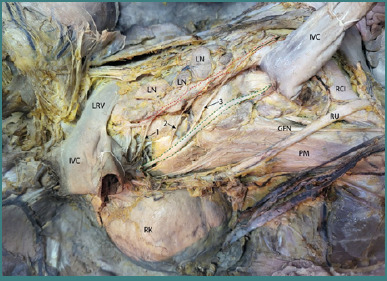
Dissection highlighting the right lumbar splanchnic nerves and the right paravertebral sympathetic chain. The approach used for this dissection was the classic anterior approach. Legend: IVC, Inferior Vena Cava; LRV, Left Renal Vein (retracted on both ends); RK, Right Kidney; PM, Psoas Major Muscle; RU, Right Ureter; GFN, Genitofemoral Nerve; LN, Preaortic Lymph Nodes; RCIA, Right Common Iliac Artery; 1 – Superior Lumbar Splanchnic Nerve; 2 – Middle Lumbar Splanchnic Nerve; 3 – Inferior Lumbar Splanchnic Nerve. Markers: Inside the red dotted line – Bundle of fibers from the right side of the aortic plexus; Inside the green dotted line – Sympathetic paravertebral chain; Black Arrow – Fibers to the superior hypogastric plexus.

Following the removal of the inferior vena cava, the lumbar vertebrae and the proximal origins of the psoas major muscle were exposed, allowing for a more detailed examination of the paravertebral sympathetic chain (Figure 4). The right paravertebral sympathetic chain can be observed descending parallel to the vertebral column, positioned anterior to the origins of the psoas major muscle. This anatomical arrangement highlights the spatial relationship between the sympathetic chain and adjacent musculoskeletal structures and may explain the possibility of injuries during extensive spinal surgery.

Three lumbar splanchnic nerves emerge from the right sympathetic chain (highlighted by the blue dotted line), coursing anteromedially toward the prevertebral aortic plexus. The three lumbar splanchnic nerves contribute to the formation of the prevertebral aortic plexus by constituting a group of preaortic fibers on the right side (highlighted by the black dotted line). Together with the left-sided preaortic fiber group, they participate in the formation of the superior hypogastric plexus at the level of the aortic bifurcation (the red arrow illustrates the contribution of the right-sided fiber group to the formation of the superior hypogastric plexus). It is also important to notice the close anatomical relation between the aortic plexus and the inter aortocaval lymph nodes, which can be clinically significant due to its implications in tumor spread, surgical procedures, and autonomic function.

**Figure 5:**
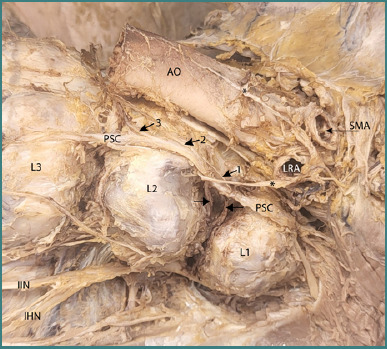
Dissection highlighting the left lumbar splanchnic nerves, the paravertebral sympathetic chain, and the communicating branches originating from the chain. The access route for this dissection was the classic anterior approach. Legend: AO, Aorta (cut); * – Efferent fibers from the left celiac ganglion; L1 – L1 vertebral body; L2 – L2 vertebral body; L3 – L3 vertebral body; SMA, Origin of the superior mesenteric artery; LRA, Origin of the left renal artery; PSC, Paravertebral sympathetic chain; IHN, Iliohypogastric nerve; IIN, Ilioinguinal nerve; 1 – Superior lumbar splanchnic nerve; 2 – Middle lumbar splanchnic nerve; 3 – Inferior lumbar splanchnic nerve. Arrows: Rami communicans.

In Figure 5, the removal of the left psoas major muscle provides a clear view of the paravertebral sympathetic chain and the origins of the left lumbar splanchnic nerves at this level. The three lumbar splanchnic nerves on the left side are observed converging toward the left preaortic bundle of nerve fibers, contributing to the aortic plexus. Furthermore, the anatomical relationship between the paravertebral sympathetic chain and the lumbar somatic plexus is distinctly highlighted, demonstrating the role of the white and gray rami communicans in facilitating neural connections between the autonomic and somatic nervous systems.

Based on the dissection findings illustrated across Figures 1 through 5, bilateral celiac ganglia were consistently identified in all specimens. In each case, their efferent fibers formed connections with the aortico-renal plexus and contributed to the preaortic network. The preaortic plexus demonstrated a consistent longitudinal organization along the abdominal aorta, with contributions from both right and left lumbar splanchnic nerves. All specimens exhibited three lumbar splanchnic nerves per side, arising from the sympathetic chain and converging toward the preaortic plexus. These findings reflect a high degree of anatomical consistency among the studied specimens.

## DISCUSSION

### Historical perspective

A brief historical perspective is relevant to appreciate the evolution of current anatomical terminology. Over time, terminology has varied significantly. For example, in Testut’s works, the celiac plexus was referred to as the *solar plexus*, which received afferents from the greater thoracic splanchnic nerve (then known as Chaussier’s greater suprarenal nerve) and the lesser thoracic splanchnic nerve (Chaussier’s lesser suprarenal nerve), and included the semilunar ganglia. Additionally, the inferior or abdominal splanchnic nerve was historically described as Walther’s posterior renal nerve [6].

The solar plexus was described alongside the aorticorenal ganglia, the superior mesenteric ganglia, and the celiac plexus itself, which extended over the celiac trunk branches and the origin of the superior mesenteric artery. Inferior to this region, Testut identified the lumboaortic plexus, and in the pelvis, he described the hypogastric plexus. Latarjet preferred the term *presacral nerve* for this latter structure, which included fibrous ganglionic masses continuing from the lumboaortic plexus and dividing into the right and left hypogastric nerves, ultimately forming the hypogastric plexus [6].

According to historical sources, the prevertebral (or splanchnic) ganglia of the thorax, abdomen, and pelvis are not discrete, isolated formations but networks of interconnected ganglionic masses. This gave rise to the more accurate term *ganglionic plexuses* [6].

### Anatomical findings and interpretation

Since there are three types of splanchnic nerves, confusion can easily arise from the use of the term 'splanchnic'. To avoid such misunderstandings, we aimed to clarify the following: the three thoracic splanchnic nerves—greater, lesser, and inferior—serve as afferents to the celiac [7] and aortico-renal ganglia [8,9]. The superior, middle, and inferior lumbar splanchnic nerves are efferents of the paravertebral chains, terminating in the prevertebral aortic plexus [10]. The sacral splanchnic nerves originate from the lateral horns of the sacral spinal cord [11], emerge from the sacral paravertebral sympathetic chains, and enter the inferior hypogastric plexus [12].

The celiac ganglia serve as the principal source of fibers contributing to the formation of the prevertebral aortic plexus. Within these ganglia, the second-order neuron (neuron II) of the sympathetic pathway is located, whereas the first-order neuron (neuron I) resides in the lateral horn of the thoracic spinal cord. This hierarchical organization highlights the central role of the celiac ganglia in the autonomic signal transmission to the abdominal viscera [13]. Also, a segmental organization delineating the precise topographical distribution of sympathetic innervation to the abdominal viscera can be noticed. Specifically, for the greater thoracic splanchnic nerve, the first-order neuron (neuron I) is located in the lateral horn of the spinal cord between T5 and T9, with its fibers terminating in the lateral portion of the celiac ganglion. In the case of the lesser thoracic splanchnic nerve, neuron I is situated at T9–T10, and its fibers project to the aortico-renal and superior mesenteric ganglia. The least splanchnic nerve originates from T12, with its fibers terminating in the renal plexus [14]. Thus, the sympathetic innervation of most abdominal organs follows a two-neuron pathway consisting of a thoracic preganglionic neuron and an abdominal postganglionic neuron, ensuring precise autonomic regulation of visceral functions.

The prevertebral ganglia are classified into three distinct groups: the celiac, superior mesenteric, and inferior mesenteric ganglia [15]. The celiac ganglia receive afferent fibers primarily from the greater thoracic splanchnic nerve, while the superior mesenteric ganglia are innervated by fibers from the lesser thoracic splanchnic nerve. In contrast, the inferior mesenteric ganglia receive input from the first two lumbar splanchnic nerves [16,17].

The lumbar splanchnic nerves, typically three, originate from the paravertebral sympathetic chain and contribute to the autonomic regulation of abdominal and pelvic organs. The superior lumbar splanchnic nerve arises at L1, the middle lumbar splanchnic nerve at L2, and the inferior lumbar splanchnic nerve at L3 [18]. The paravertebral ganglia serve as the site for the second-order neuron (neuron II) of the sympathetic pathway, while the first-order neuron (neuron I) is situated in the lateral horn of the spinal cord at the L1–L2 level. Based on these observations, we can note that their anatomical arrangement is in accordance with the hierarchical organization of the rest of the sympathetic nervous system.

The lumbar splanchnic nerves contribute to the formation of the prevertebral aortic plexus, giving rise to two distinct preaortic fiber groups—one on the right and one on the left. These fiber groups converge at the level of the aortic bifurcation, where they merge to form the superior hypogastric plexus, providing an important link between the abdominal and pelvic sympathetic systems. These findings align with recent research by Pontecorvo *et al*., who provided detailed insights into the variability and clinical implications of the lumbar sympathetic trunk, emphasizing its relevance in surgical procedures involving the retroperitoneal space [19].

### Clinical perspective

The abdominal sympathetic structures, deeply embedded within the retroperitoneal space, present significant challenges for surgical access due to their proximity to major vascular structures. Any attempt to manipulate or resect these neural formations risks iatrogenic injury, which may lead to vascular damage, severe hemorrhage, or autonomic dysfunction [20,21].

One clinically relevant aspect of the preaortic sympathetic plexus is its relationship with retroperitoneal lymph nodes, particularly the interaortocaval lymph nodes, which serve as key drainage points for abdominal organs. Lymphadenopathy, whether due to malignancy, infection, or inflammatory conditions, may compress the aortic plexus, leading to neuropathic pain, vascular compromise, or autonomic disturbances such as altered gut motility and urinary and erectile dysfunction [22,23]. This underscores the importance of careful dissection techniques in retroperitoneal lymphadenectomy, particularly in cases of testicular cancer, lymphoma, or other metastatic diseases [24].

Moreover, surgical procedures involving the abdominal aorta, including aneurysm repair, endovascular interventions, and aortoiliac bypass, must consider the delicate neural networks within the preaortic plexus. Disruption of these fibers can result in chronic visceral pain syndromes or functional impairments, such as postoperative ileus or impaired ejaculation due to superior hypogastric plexus injury [25].

From a diagnostic perspective, understanding the preaortic sympathetic plexus is essential in radiological imaging, as MRI and CT scans may reveal pathologic involvement of these structures in cases of retroperitoneal fibrosis, neurogenic tumors, or sympathetic dysfunction syndromes [26,27]. Clinicians should recognize that abnormal sympathetic activity in this region may present with chronic abdominal pain, unexplained gastrointestinal symptoms, or orthostatic hypotension, necessitating targeted evaluation and possible sympathetic blockade procedures [28,29].

Finally, nerve-sparing techniques in retroperitoneal surgery have gained increasing importance. Minimally invasive approaches, such as laparoscopic and robotic-assisted procedures, may reduce the risk of inadvertent sympathetic nerve injury, preserving autonomic function while maintaining oncological and vascular surgical goals [30].

Beyond its anatomical complexity, the preaortic plexus has important physiological roles. Although the primary objective of this study was anatomical, the functional relevance of the preaortic sympathetic plexus must be emphasized. This plexus plays a central role in the autonomic regulation of gastrointestinal motility, renal perfusion, and gonadal function. Disruption of its pathways—whether through surgery, trauma, or pathology—can result in various autonomic dysfunctions, including delayed gastric emptying, altered intestinal transit, and even impaired ejaculation due to the involvement of the superior hypogastric plexus. Therefore, understanding the detailed anatomy of this plexus has direct implications for preserving function during retroperitoneal and pelvic surgeries. Future studies incorporating neurophysiological or immunohistochemical techniques could further elucidate the functional organization of this network.

### Study limitations and acknowledgments

The primary objective of this study was descriptive, and as such, no quantitative statistical analysis of anatomical variability was performed. However, all dissections consistently revealed the presence of three lumbar splanchnic nerves and the formation of a preaortic plexus, supporting the reproducibility of the main findings.

While this study was focused on dissection-based anatomical observations, the authors acknowledge the importance of correlating these findings with radiological anatomy to enhance clinical applicability. Imaging modalities such as MRI and CT play an essential role in evaluating retroperitoneal structures, and future studies integrating cadaveric dissection with radiologic imaging would offer valuable insights. This combined approach could improve the identification of sympathetic plexuses in preoperative planning and the assessment of pathologies such as retroperitoneal fibrosis, ganglioneuromas, or lymphadenopathy involving the preaortic region.

A limitation of this study is the relatively small number of cadavers available for dissection. While this number may seem limited in absolute terms, it reflects the current realities anatomical departments face. Following the COVID-19 pandemic, legislative changes have significantly restricted cadaver availability. At our institution, the few cadavers received must primarily serve the educational needs of medical students. Moreover, detailed dissections such as those required for this study result in the irreversible alteration of anatomical structures essential for teaching general anatomy. Therefore, a balance must be maintained between preserving cadavers for student education and dedicating them to research. We prioritized the quality and depth of each dissection, aiming to generate consistent and meaningful observations that enhance understanding of the preaortic sympathetic plexus and its clinical relevance. Despite these limitations, our findings contribute valuable anatomical insight with potential applications in surgical planning and medical education.

## CONCLUSION

The preaortic sympathetic plexus is a complex and consistent network extending from the upper lumbar region to the aortic bifurcation. In all specimens, bilateral celiac ganglia were identified as the primary source of efferent fibers, which joined those from the lumbar splanchnic nerves arising from the paravertebral sympathetic chains. These fibers formed a longitudinally oriented plexus along the anterior aspect of the abdominal aorta, contributing to the formation of secondary autonomic plexuses such as the superior and inferior mesenteric plexuses, as well as the periarterial plexuses surrounding the gonadal vessels. The renal plexuses arise as efferent fibers from the celiac ganglia. The prevertebral ganglia include the celiac, aortic, superior mesenteric, and inferior mesenteric ganglia, each associated with thoracic or lumbar splanchnic nerves. All thoracic and lumbar splanchnic nerves carry preganglionic fibers, which synapse within the prevertebral ganglia, such as the inferior mesenteric or hypogastric plexus. The lumbar splanchnic nerves, originating from the paravertebral chains, also connect with the somatic lumbar plexus via white and gray rami communicans.

The consistent identification of these structures across all dissections highlights the organized architecture of the sympathetic nervous system in the retroperitoneal space. This anatomical understanding has important clinical implications, particularly in retroperitoneal surgery, where nerve-sparing techniques are essential for minimizing autonomic dysfunction and preserving visceral function.

## Data Availability

Data is contained within the article.
